# The Annual Meetings at Niagara

**Published:** 1892-09

**Authors:** 


Adjourned.

### Editorial.

### THE ANNUAL MEETINGS AT NIAGARA.

The American Dental Association has had its annual convocation, and its work for this year has passed into history. We fear there can be but one verdict in regard to it,—that, with few exceptions, the papers presented were not equal to the standard such a body should demand. As a natural result, the discussions were equally unsatisfactory. The fault does not lie with the sections, as they did all that men could do to make this meeting a notable one, and that it was practically a failure from a scientific standpoint is chargeable upon the spirit of indifference in the profession at large.

The contrast between this meeting and that held at Saratoga Springs last year was marked, and it seemed unfortunate that it was so, in view of the fact that it appeared to have a discouraging effect upon some, and may deter them from any active participation in the more important gathering next year at Chicago. That the approaching Columbian Dental Congress absorbed the interest was clearly apparent, and led the members to regard this meeting as merely preliminary to that in Chicago next year. It is to be hoped that if this be so, the neglect of this means genuine scientific work for that international gathering.

The numbers present were fully equal to anticipation, and the social interchanges of men gathered from all parts of the country compensated in a large degree for deficiencies in other directions.

Niagara Falls as a place of meeting has superior advantages; not the least this year was the exceptional coolness, very grateful after the extreme heat of the previous week.

The Association declined to consider at this meeting the important changes suggested by the Committee on Constitution, these being laid over for next year. It is very questionable whether this was a wise decision. It seems more than probable that there will be less time and inclination to give attention to the various subjects then than would have been given them at this meeting.

The Association decided to meet next year at Chicago and confine the work entirely to routine business. It would have been, perhaps, better to have adjourned over until 1894, as it is not probable that a sufficient number of members will be willing to sacrifice the time necessary to the completion of several important matters.

The National Association of Dental Faculties was fully attended, and proved an unusually harmonious meeting. It is gratifying to note that the friction between this organization and that of the National Association of Dental Examiners, so marked last year, was not perceptible this. There seems to have been a growing effort in both organizations to harmonize differences. If this spirit be continued, there is reason to hope that some of the very objectionable laws now on the statute-books of several of the States may be modified in the near future.

The vote of censure passed on one of the most prominent dental colleges for granting an honorary degree indicated the sentiment of the organization very clearly on that important matter. This was further enforced in the application of a college for admission where three degrees of similar character had been conferred. The spirit manifested was so positive that it may be safely assumed that this degree will not be permitted in the future.

The State boards were fully represented in the National Association of Dental Examiners. The disposition to refer all disputed questions to committees of conference was a move in the right direction, and led very satisfactorily to the harmonizing of differences between the two governing bodies.

The prevailing feeling seemed to be paramount at Niagara that the meeting to be held at Chicago must mark an epoch in American dentistry, and if the interest manifested be a basis for judgment, there need be no anxiety as to the result. To make this a marked success means a year of thorough scientific work for all who propose to take part in its deliberations.

